# Challenges in the diagnosis of pulmonary mucormycosis in a diabetic with a review of literature

**DOI:** 10.1016/j.rmcr.2021.101474

**Published:** 2021-07-08

**Authors:** Muhammad Yousaf, Sarah Salameh, Irfan Ul Haq, Samir Alhyassat, Merlin Thomas, Aisha Hussain, Mohd Wani, Ehab Massad, Hamad Abdel Hadi, Hisham Abdul Sattar, Mansoor Hameed

**Affiliations:** a Hazm Mebaireek Hospital, Hamad Medical Corporation, Doha, Qatar; b Hamad General Hospital, Hamad Medical Corporation, Doha, Qatar; c Weill Cornell Medicine-Qatar, Cornell University, Doha, Qatar; d Communicable Diseases Centre, Hamad Medical Corporation, Qatar; e Department of Laboratory Medicine and Pathology, Hamad Medical Corporation, Qatar; f Department of Cardiothoracic Surgery, Hamad Medical Corporation, Qatar

**Keywords:** Mucormycosis, Zygomycosis, Invasive fungal infections, Diabetes, Pneumonia, Antifungal, Hemoptysis

## Abstract

Diabetes Mellitus appears to be the most common underlying condition associated with mucormycosis; a rare opportunistic fungal infection associated with high morbidity and mortality. Pulmonary mucormycosis may mimic pneumonia and thus pose challenges in achieving a timely diagnosis critical to successful outcomes. We present a case of a 65-year-old diabetic who presented with fever and haemoptysis that was managed as pneumonia. A bronchial alveolar lavage grew *Rhizopus* mould that was thought to be a contaminant as he responded well to antibiotics. He required another admission in 4 weeks due to worsening symptoms. Failure to respond to antibiotics and ongoing clinical and radiological deterioration led to a lobectomy that confirmed a diagnosis of pulmonary mucormycosis. He responded well to surgical resection and antifungal therapy with a complete recovery. Elusive clinical presentation and insensitive conventional diagnostic techniques may make the diagnosis of mucormycosis challenging. Our case reports highlight the issues involved in the diagnosis and management of pulmonary Mucormycosis mimicking as pneumonia.

## 1 Introduction

The burden of invasive fungal infections (IFI) has significantly increased over the last three decades due to an increase in immunosuppression, solid organ transplants and HIV epidemic [1]. Mucormycosis is a deadly angioinvasive fungal infection particularly common among diabetics, haematological malignancies [2]. It is the fourth most common opportunistic fungal infection after candidiasis, aspergillosis and dimorphic fungi [3]. The most common condition associated with mucormycosis is diabetes (36%) followed by haematological malignancies (19%) [1,3]. After aspergillosis, it is the second most common fungal infection in those with haematological malignancies or those who have had either a haematopoetic stem cell transplant or solid organ transplant [4]. These invasive fungal infections (IFI) have high morbidity and mortality and a proactive approach to diagnosis and treatment is critical for successful outcomes [5]. We report a case of pulmonary mucormycosis in a diabetic patient and discuss diagnostic challenges due to non-specific presentation of pulmonary mucormycosis and the effectiveness of combined modality treatment in mucormycosis.

### 1.1 Case report

A 65-year-old Egyptian gentleman presented with fever, cough and hemoptysis for 1 week. The hemoptysis occurred 4–6 times a day and he coughed up approximately a teaspoon of red blood each time. There was no chest pain or shortness of breath. He had a low-grade fever and possibly some weight loss over the past four weeks. A systematic review did not yield any other significant symptoms.

He was an ex-smoker with a history of 30 pack years. His past medical history included diabetes, isolated right abducent nerve palsy and chronic hepatitis C with mild hepatomegaly. There was no history of tuberculosis (TB) or contact with TB. There was no family history of any note. His current medications included aspirin and Atorvastatin and metformin. On admission, his vital signs showed a temp of 38 °C, RR 14/min, BP 108/80 mm (Hg) and SpO2 95% on room air. He had decreased air entry and dullness to percussion on the right apical chest area. His weight on admission was 70Kg with a BMI of 25. A chest X-ray on admission showed widened mediastinum with right apical infiltrates. Baseline blood tests showed normal WBC count, coagulation profile and platelet count with mild anaemia of Hb 11.8 gm/dl. The CRP was marginally raised at 16mg/L with a normal procalcitonin. He also had slightly raised total bilirubin 33umol/L and AST of 55 U/L with a normal ALT. Blood cultures and sputum workup for tuberculosis was negative. As part of his investigation a Human immunodeficiency virus (HIV) PCR was also sent that came back as negative. He had a poorly controlled Diabetes at presentation as indicated by an HbA1c of 16.6%, however, with appropriate management a repeat HbA1c at 3 months improved to 6.5%.

**Image 1:**
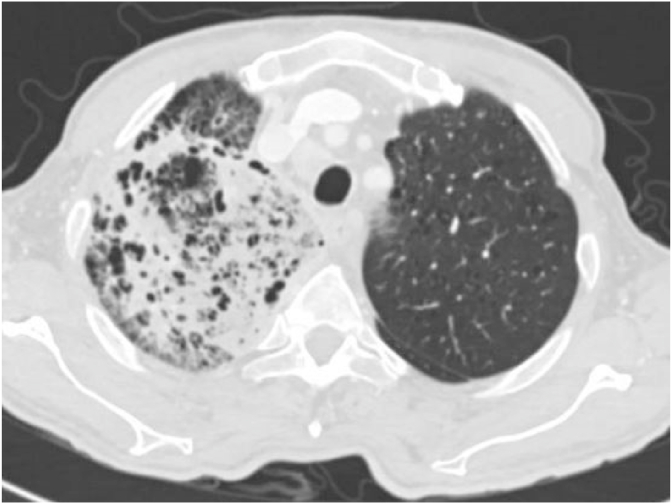
CT Chest showing extensive ground glass and cystic changes in right upper lobe.

On day 3, a CT scan of the chest showed extensive right upper lobe cystic and consolidatory changes with multiple enlarged mediastinal lymph nodes (Image 1). On Day 7, he had a bronchial alveolar Lavage (BAL) that did not show evidence for bacterial infection or malignancy. The gram stain, cultures and TB workup were negative. The BAL fluid was predominantly neutrophilic (96% neutrophils) and grew Rhizopus species. The clinical impression was that of bacterial pneumonia based on the acuteness of illness and neutrophilic predominant BAL fluid. The presence of Rhizopus in BAL was thought to be a contaminant as he had a good response to ceftriaxone. The temperature spikes settled with good clinical response, and he was discharged on oral Augmentin for further 2 weeks. A pulmonology clinic follow up was arranged for 6 weeks.

However, 4 weeks later, he presented to the emergency department with dizziness, hemoptysis and significant weight loss (10 Kg). An MRI brain did not reveal anything significant. He did not have a fever and vital signs were normal with 100% oxygen saturation on room air. Blood results showed a normal white cell count and CRP. A chest X-ray showed progression in consolidation compared to the last admission.

A repeat CT scan of the chest revealed a newly developed cavity in the right upper lobe with an air-fluid level suggestive of a lung abscess. He was started on ceftriaxone and metronidazole for lung abscess, however due to the poor response to antibiotics, a CT guided diagnostic aspiration of lung abscess was carried out. The aspirated blood-stained fluid sample sent to the lab did not show any evidence for infection or malignancy.

**Image 2:**
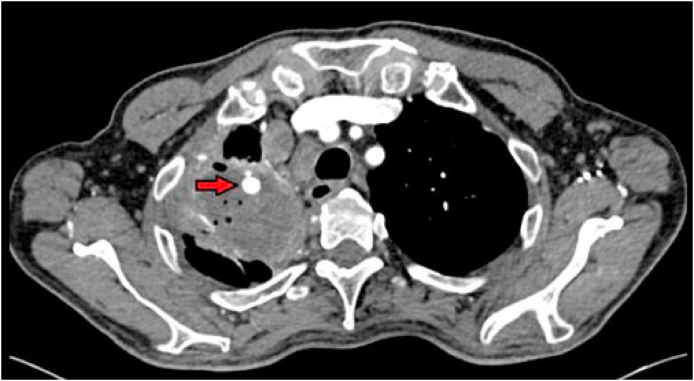
Pulmonary artery aneurysm noted within right upper lobe cavity.

The CT scan also showed an aneurysm measuring 8 × 7mm within the cavity which appeared to arise from a subsegmental branch of the right upper lobe pulmonary artery (image 2). Later, he had a successful pulmonary artery embolization due to ongoing hemoptysis.

**Image 3:**
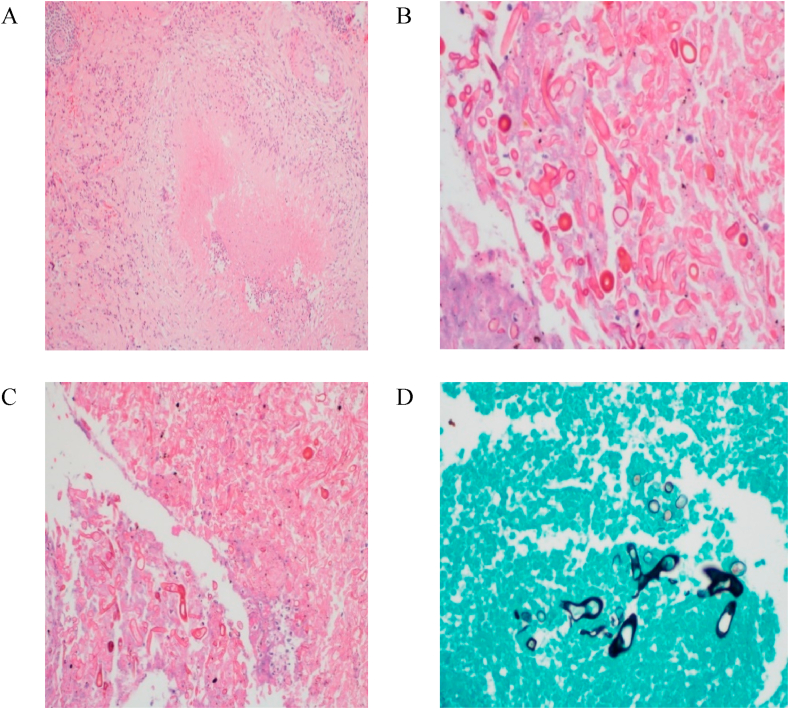
**Histological appearance**. Fungal hyphae identified at high magnification with PAS special staining (Fig 4 A, B & C). The fungi are wide, 10–15 μm in diameter, variable in width, and branching at right angles (Fig. 4 D: Grocott special stain), confirming the diagnosis of Mucormycosis.

Despite 3 weeks of antibiotics, hemoptysis continued, and radiology failed to improve. Thus, Thoracic surgeons were consulted, who successfully performed a right upper lobe lobectomy. The pathology report post-surgery showed caseating granulomatous inflammation and septated thick wide fungal hyphae, branching at a right angle, consistent with mucormycosis (Image 3). There was no evidence of malignancy or tuberculosis. A diagnosis of Pulmonary mucormycosis was made and he was given a 2-week course of amphotericin followed by another 2 weeks of posaconazole. He made an excellent recovery post-surgery and was successfully discharged home. On outpatient follow up, he continued to improve and regained his weight as well.

## 2 Discussion

We present a case of pulmonary mucormycosis (PM) in a diabetic who had a rather late diagnosis of mucormycosis, however, a successful outcome as a result of combined modality treatment. We discuss challenges in the diagnosis and management of pulmonary mucormycosis and its association with diabetes mellitus.

Mucormycosis is a rare invasive fungal infection associated with high mortality [4] and caused by a variety of moulds, *Rhizopus* being the most frequent cause (65%) [6]. Mucormycosis occurs almost exclusively either in diabetics or in the immunocompromised [4]. A meta-analysis of mucormycosis case reports by Jeong et al. showed diabetes to be the most common underlying condition (40%) for mucormycosis [4]. Diabetes also seems to be an independent risk factors for mucormycosis among hematologic malignancies, immunocompromised and solid organ transplant recipients [7]. The pathophysiology of diabetes-associated mucormycosis is not fully understood but it is reported to be possibly linked to neutrophilic dysfunction and reduced phagocytic effect of macrophages secondary to low pH, ketones and hyperglycaemia [7,8]. Mucorales virulence appears to be linked to its ability to acquire iron from the host and the combination of hyperglycemia and acidic pH in diabetic ketoacidosis (DKA) seems to facilitate iron uptake by Mucorales, making DKA particularly susceptible to Mucormycosis [9].

Our case had a rather late diagnosis of PM as it was initially managed as acute pneumonia that responded well to antibiotics and in that context, a BAL sample that grew *Rhizopus* was thought to be a contaminant. Pulmonary mucormycosis may mimic pneumonia and that combined with a low index of clinical suspicion will often delay the diagnosis till a patient fails to respond to conventional antibiotics [10]. Other factors that may lead to a delayed diagnosis includes concomitant bacterial or viral infections (39% cases), positive fungal results considered as a contaminant and invasive tests required to obtain an adequate sample [10].

Imaging in PM is usually non-specific, as in our case, though a reverse halo sign (RHS) on a CT chest may point towards a diagnosis of PM rather than invasive aspergillosis. A study of 189 patients by Hisham et al. showed RHS to be present only in 4% of cases of invasive fungal infections, however, higher prevalence in PM than invasive aspergillosis (8% Vs 1% respectively) [11]. Since RHS may present in a variety of infectious or non-infectious conditions, therefore, its significance may depend upon a high pre-test clinical probability [12]. Our case had a pulmonary artery aneurysm which is a rare radiological finding in PM. Pulmonary aneurysm when occurs adjacent to or within a cavity are often termed as Rasmussen aneurysm (Image 2) [13].

It is vital to recognise host factors and clinical manifestation of mucormycosis in a typical clinical scenario and act promptly to confirm the diagnosis with imaging modalities and acquisition of samples for histopathological confirmation [14]. Like in our case, the team should have been more proactive in considering a diagnosis of fungal infection on the background of diabetes, *Rhizopus* in BAL and clinical deterioration despite initial good response to conventional antibiotics. We have summarised clinical features, clues and strategies to aid in the diagnosis of mucormycosis in Table 1.

**Table 1:** Clues to proactively diagnose mucormycosis.

1. High index of clinical suspicion in appropriate clinical settings (*see below*) [14]
2. Prompt imaging and being proactive with invasive testes to get adequate samples [10]
3. Histopathology and cultures are the cornerstone of the diagnosis [19,23].
4. Histopathology appears to be more sensitive than fungal cultures [16].
5. Be cautious labelling positive microbiological samples for fungus as “contaminant” in appropriate clinical scenarios or if failure to respond to antibiotics [10].
6. Commonest species are Rhizopus (48%) and Mucor (14%) [4].
7. Imaging: Predilection for upper lobes particularly right upper lobe [16].
8. CT chest reverse halo sign observed in 4% cases only but may differentiate PM from aspergillosis [12].
9. Serology Tests e.g., galactomannan and 1,3-β-*d*-glucan assay may help differentiate mucormycosis from other fungi like aspergillus [19].
**Clinical Settings/Factors that should raise suspicion for mucormycosis**
**Pulmonary Mucormycosis**: Commonly present as localised disease (54%) mimicking as pneumonia; fever (63%), cough (61%) haemoptysis (26%) commonest symptoms [4,16].
**A Diabetic with diplopia** may be a clue to Rhino-orbital-cerebral mucormycosis [22].
**Neutropenia** is an important predisposing factor [4].
Mucormycosis can occur **in non-immunocompromised** e.g., Diabetes Mellitus
**Host Factors** predisposing to Mucormycosis: Diabetes Mellitus (40%) and Haematological malignancies (33%) [4,6].
**Commonest sites involved**: Sinus/Rhino-orbital-cerebral (39%), Pulmonary (24%) and Cutaneous (19%) [6].

Mucormycosis is associated with angioinvasion and extensive necrosis and thus a combination of medical and surgical modalities appears to have better survival than the medical therapy alone [10,[15], [16], [17]]. A study by Choi et al. compared combination therapy against medical therapy alone; they reported 9 of 11 patients who underwent both surgical and medical therapy survived while only 1 of 9 who had antifungal therapy alone survived, showing a striking benefit of combined modality treatment that has been supported in other studies [16,17]. There is no clear guidance on the timing of surgery but should be considered within 72 hours if one fails to respond to appropriate antifungal therapy as progressive dissemination of disease may make surgery less beneficial [10]. Lobectomy, as in our case, remains the most common procedure and pneumonectomy reserved for the more proximal or extensive disease [15].

We managed our case with amphotericin B and Posaconazole post-operatively. Amphotericin B has been established over the years as a standard first-line antifungal agent though there are no randomised clinical efficacy trials [18,19]. Antifungal therapy should be prompt to improve survival outcomes; Chamilos et al. showed that a delayed amphotericin B therapy (>6 days from diagnosis) was associated with a 2-fold increase in mortality [20]. There is no consensus on the duration of treatment but ideally should be continued until there are clinical or radiological resolution and reversal of immunosuppression [21]. Good glycaemic control in diabetics, as in our case, is vital not only to the management of mucormycosis but also in its prevention [22].

## 3 Conclusion

Recognition of host factors and a high index of clinical suspicion in an appropriate clinical scenario is the key to prompt diagnosis and management of mucormycosis. Optimal treatment of mucormycosis requires an early institution of therapy via a multidisciplinary approach to improve survival outcomes in this deadly invasive fungal infection.

## Ethics statement

The study was approved by the Institutional Review Board of Hamad Medical Corporation (MRC-04-21-072).

## Financial support

The publication of this article is supported by the Qatar National Library, Qatar.

## Declaration of competing interest

The authors declare that they have no known competing financial interests or personal relationships that could have appeared to influence the work reported in this paper.
